# Sex differences in DNA methylation variations according to ART conception-evidence from the Norwegian mother, father, and child cohort study

**DOI:** 10.1038/s41598-024-73845-3

**Published:** 2024-10-02

**Authors:** Dana Kristjansson, Yunsung Lee, Christian M. Page, Håkon Gjessing, Maria C. Magnus, Astanand Jugessur, Robert Lyle, Siri E. Håberg

**Affiliations:** 1 Center for Fertility and Health, Norwegian Institute of Public Health, Skøyen, Postboks 222, 0213 Oslo, Norway; 2 Department of Genetics and Bioinformatics, Norwegian Institute of Public Health, Oslo, Norway; 3 Department of Physical Health and Ageing, Division of Mental and Physical Health, Norwegian Institute of Public Health, Oslo, Norway; 4 Department of Global Public Health and Primary Care, University of Bergen, Bergen, Norway; 5 Department of Medical Genetics, Oslo University Hospital, Oslo, Norway

**Keywords:** ART, Assisted reproductive technologies, DNA methylation, Sex differences, Fertility, Reproduction, Epigenetics, DNA methylation, Infertility

## Abstract

Previous studies have shown cord-blood DNA methylation differences in newborns conceived using assisted reproductive technologies (ART) compared to those conceived naturally. However, whether these ART-related DNA methylation differences vary with children’s sex is unknown. We hypothesize that the DNA methylation differences in cord blood between ART-conceived and naturally conceived newborns also varies by the sex of the child, with distinct patterns of differential methylation present in males and females. We investigated sex differences in cord-blood DNA methylation variation according to conception by ART using the Illumina MethylationEPIC platform, comparing 456 ART-conceived versus 507 naturally-conceived girls, and 503 ART-conceived and 473 naturally-conceived boys. We identified 37 differentially methylated CpGs according to ART-conception among girls, and 70 differentially methylated CpGs according to ART-conception among boys, when we used a 1% false discovery rate to account for multiple testing. Ten CpGs were differentially methylated according to conception by ART in both sexes. Among the genes that were associated with these CpGs, we found the BRCA1; NBR2 gene (two CpGs) was hypermethylated in girls while the APC2 (two CpGs) and *NECAB3;ACTL10*, (four CpGs) related to cellular signaling were hypomethylated in boys. These findings confirm the presence of sex-specific epigenetic differences, illustrating the nuanced impact of ART on the fetal epigenome. There is a need for further explorations into the implications for sex-specific developmental trajectories and health outcomes in ART-conceived children.

**Supplementary Information:**

The online version contains supplementary material available at 10.1038/s41598-024-73845-3.

## Introduction

The prevalence of newborns conceived through assisted reproductive technologies (ART) varies across different countries, ranging from 1.2 to 6.3%^1^. As the global proportion of children conceived by ART increases^1^, understanding the potential health consequences of these technologies becomes increasingly important.

ART involves the chemical and physical manipulation of gametes and embryos under conditions that differ from normal physiological conditions^2^, which may introduce factors that could influence the development and health of the offspring^3^. Epigenetic modifications, gene activity, cell function, and development, have been the focus of several investigations, especially because ART involves manipulating and culturing early embryos during a critical period of extensive epigenetic reprogramming^4^. Since DNA methylation (DNAm) is stable over time, analyzing CpGs in newborns may offer valuable insights into the potential long-term health consequences of ART^5,6^.

Offspring conceived by ART have an elevated risk of experiencing adverse perinatal outcomes such as lower birth weights, cerebral palsy, and respiratory defects^7^. There is also emerging evidence of longer-term health effects, including altered glucose metabolism^8^, elevated blood pressure^8,9^, and compromised cardiac function such as alteration of systolic function and long-term pediatric cardiovascular disease^10,11^. While many studies involve small sample sizes and there is a lack of studies investigating sex differences in health outcomes in ART-conceived children, an extensive body of research demonstrates that ART-conceived offspring exhibit distinct DNAm differences compared to naturally-conceived offspring. These differences are evident in several genes associated with growth^12,13^, neurodevelopment^13,14^, and cancer^13^, —conditions known to have clear sex differences. Furthermore, early-life health traits such as birthweight^15^, neurodevelopmental disorders^16,17^, cleft lip^18,19^, and cancers^20^, have long been shown to differ by sex. Our previous study on parental methylomes and their children had already found that associations in ART-conceived children compared to naturally-conceived children persisted after controlling for parents’ DNA methylation and are not explained by parental subfertility^13^. Investigating potential sex differences in ART methylation differences is crucial as it may provide insights into the underlying mechanisms contributing to the observed health disparities among ART-conceived children.

As the embryonic development is sex-specific, and DNAm is essential for embryonic development, many epigenetic marks identified at birth are found to be sex-specific^21^. While studies have documented sex differences in birth outcomes among ART-conceived infants^19,22^, the extent to which ART influences DNAm in a sex-specific manner remains unclear. Uncovering these differences is crucial because ART may impact health outcomes and may vary between girls and boys.

In this study, we investigated sex-specific differences in cord-blood DNAm variation according to conception by ART among 959 ART-conceived (456 girls and 503 boys) and 980 naturally-conceived newborns (507 girls and 473 boys) using the Illumina EPIC platform.

## Results

**Fig. 1:**
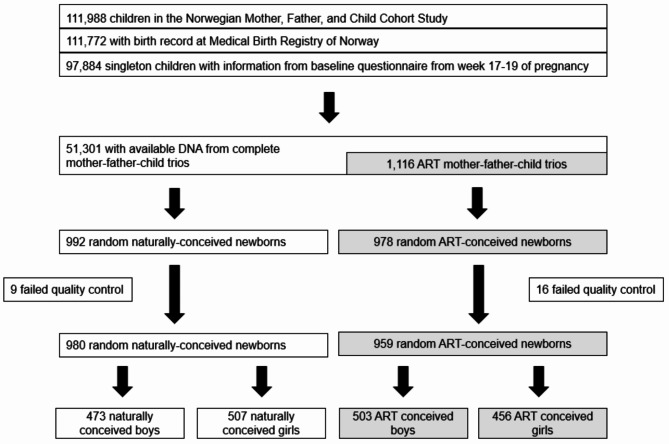
Selection of study participants.

The children in this study were the result of pregnancies in the Norwegian Mother, Father and Child Cohort Study (MoBa) during the enrollment period of 1999 to 2008^23^. Following data processing and quality control, DNAm data from the Illumina MethylationEPIC array were available for 963 girls (456 ART-conceived and 507 naturally-conceived) and 976 boys (503 ART-conceived and 473 naturally-conceived) (Table 1; Fig. 1). These data covered 770,586 autosomal CpGs for each individual in the final data set (refer to “Methods” for details).

**Table 1:** Characteristics of the study participants.

Characteristics	Boys (*n* = 976)		*P* value^a^	Girls (*n* = 963)		*P* value^a^
	Naturally-conceived	ART-conceived		Naturally-conceived	ART-conceived	
Natural vs. ART conceived, No.	473	503		507	456	
Child gestational age, mean (SD)	39.7 (1.6)	39.4 (1.8)	0.02	39.4 (1.6)	39.5 (1.5)	0.54
Missing, No. (%)	2 (0.43)	16 (1.4)		2 (0.4)	15 (3.3)	
Child birthweight, g, mean (SD)	3732.5 (540.0)	3547.7 (567.7)	< 0.001	3571.3 (499.1)	3502.8 (506.1)	0.03
Missing, No. (%)	0 (0)	1 (0.2)		0 (0)	0 (0)	
Time to pregnancy, No. (%)						
No information	4 (1.0)	68 (13.7)	< 0.001	6 (1.5)	45 (10.0)	< 0.001
	251 (63.1)	64 (12.9)		278 (67.0)	56 (12.5)	
3–8 months	61 (15.4)	9 (1.8)		67 (16.1)	12 (2.7)	
9–12 months	38 (9.6)	23 (4.7)		28 (6.8)	20 (4.5)	
	43 (10.8)	331 (66.9)		36 (8.7)	316 (70.4)	
Type of ART, No. (%)						
Fresh embryo transfer IVF	NA	224 (44.5)		NA	212 (46.5)	
Fresh embryo transfer ICSI	NA	177 (35.2)		NA	148 (32.5)	
Frozen embryo transfer IVF	NA	44 (8.8)		NA	43 (9.4)	
Frozen embryo transfer ICSI	NA	22 (4.4)		NA	17 (3.7)	
Combination/unspecified	NA	34 (6.8)		NA	3 (0.7)	
Maternal age at delivery, years, mean (SD)	30.0 (4.6)	33.2 (3.6)	< 0.001	30.0 (4.6)	33.2 (3.7)	< 0.001
Paternal age at delivery, years, mean (SD)	32.8 (5.5)	35.7 (5.5)	< 0.001	32.5 (5.4)	35.9 (5.3)	< 0.001
Maternal parity, No. (%)						
Nulliparous	218 (46.1)	355 (70.6)	< 0.001	243 (47.9)	317 (69.5)	< 0.001
Multiparous	255 (53.9)	148 (29.4)		264 (52.1)	139 (30.5)	
Maternal educational level, No. (%)						
Less than high school	38 (8.0)	25 (5.0)	0.03	32 (6.3)	23 (5.0)	0.08
High school	137 (29.0)	121 (24.1)		160 (31.6)	110 (24.1)	
Up to 4 years of college	188 (39.8)	221 (43.9)		198 (39.1)	196 (43.0)	
More than 4 years of college	109 (23.0)	131 (26.0)		116 (22.9)	126 (27.6)	
Missing	1 (0.2)	5 (1.0)		1 (0.2)	1 (0.2)	
Paternal educational level, No. (%)						
Less than high school	53 (11.2)	35 (7.0)	0.04	44 (8.7)	37 (8.1)	0.04
High school	188 (39.8)	186 (37.0)		208 (41.0)	146 (32.0)	
Up to 4 years of college	122 (25.8)	150 (29.8)		127 (25.1)	145 (31.8)	
More than 4 years of college	91 (19.2)	118 (23.5)		118 (23.3)	117 (25.7)	
Missing	19 (4.0)	14 (2.8)		10 (2.0)	11 (2.4)	
Maternal pre-pregnancy BMI, kg/m², mean (SD)	24.0 (4.2)	24.1 (3.9)	0.70	24.2 (4.1)	24.3 (3.9)	0.72
Missing, No. (%)	12 ( 2.5)	11 (2.2)		11 (2.2)	9 (2.0)	
Paternal pre-pregnancy BMI, mean (SD)	25.8 (3.2)	26.3 (3.6)	0.02	26.0 (3.3)	26.5 (3.3)	0.03
Missing, No. (%)	19 (4.0)	18 (3.6)		23 (4.53)	14 (3.1)	
Maternal smoking during pregnancy, No. (%)						
Never	228 (48.2)	260 (52.0)	< 0.001	262 (52.7)	233 (51.1)	< 0.001
Former	127 (26.9)	178 (35.4)		125 (24.7)	179 (39.3)	
Quit before 18 gestational weeks	59 (12.5)	41 (8.2)		73 (14.4)	21 (4.6)	
Continued after 18 gestational weeks	56 (11.8)	23 (4.6)		46 (9.1)	20 (4.4)	
Missing	3 (0.6)	1 (0.2)		1 (0.2)	3 (0.7)	
Paternal smoking, No. (%)						
No	350 (74.0)	377 (75.0)	0.86	395 (77.9)	358 (78.6)	0.3
Yes	121 (25.6)	123 (24.6)		112 (22.1)	96 (21.1)	
Missing	2 (0.4)	3 (0.6)		0 (0)	2 (0.4)	

*SD* standard deviation, *BMI* body mass index, *IVF* in vitro fertilization, *ICSI* intracytoplasmic sperm injection.

^a^*P* values from one sided chi-square tests (categorical variables) or two-sided t-test (continuous variables).

### DNA methylation in newborn girls and boys

**Fig. 2:**
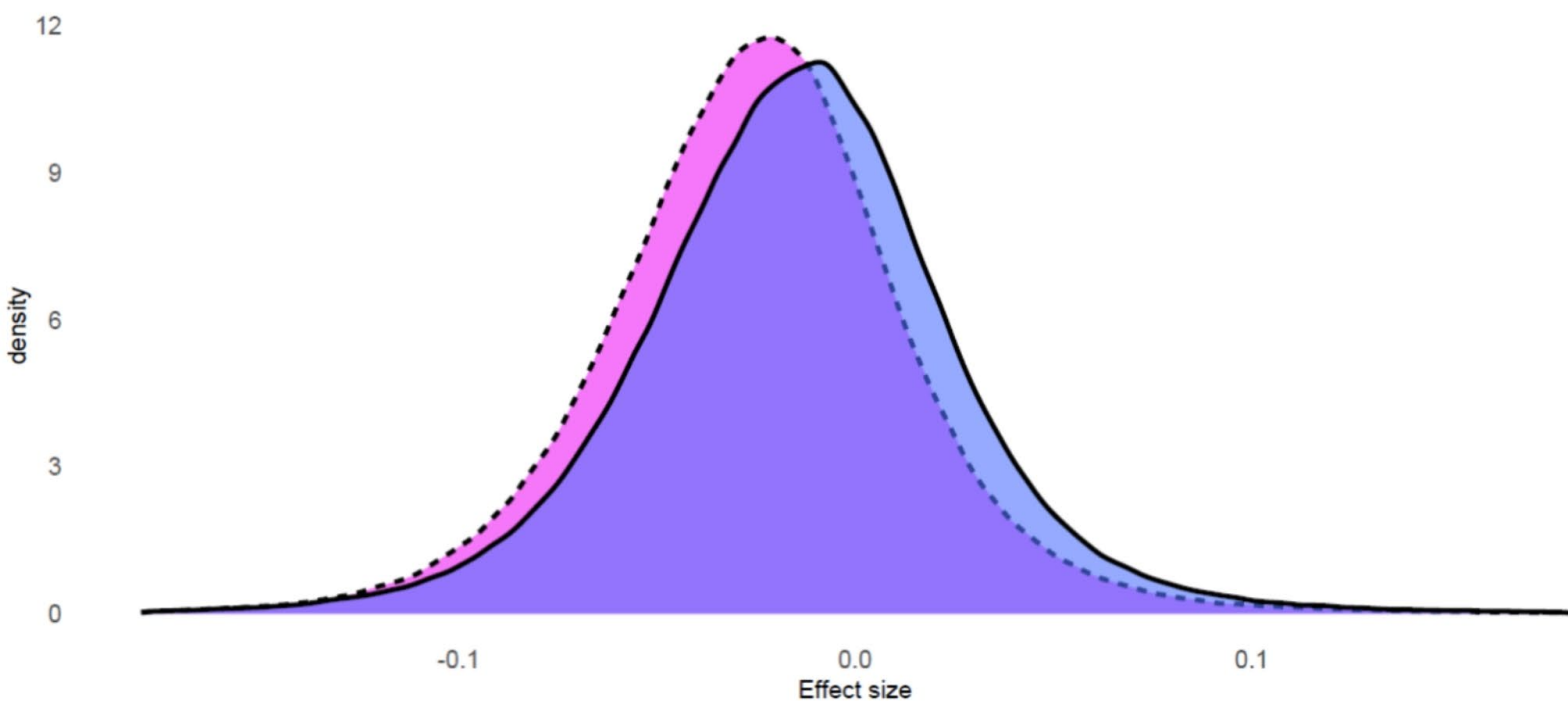
Differences in the distribution of genome-wide DNA methylation between ART-conceived and naturally-conceived girls (pink with dotted line), and between ART-conceived and naturally-conceived boys (blue with solid line).

In terms of DNAm, both ART-conceived girls and boys exhibited an overall lower genome-wide methylation than their naturally-conceived counterparts. The differences were more pronounced in ART-conceived girls compared to naturally-conceived girls than in ART-conceived boys compared to naturally-conceived boys (Fig. 2), with a Kolmogorov-Smirnov (KS) test value of 0.118 ( *P* < 2.2 × 10^− 16^), indicating significant differences between boys and girls.

After adjusting for factors such as maternal age, maternal smoking, maternal BMI, child sex, parity, and plate ID, we identified significant differences in DNAm according to conception by ART in both sexes. Specifically, 37 CpGs were differently methylated according to conception by ART in newborn girls and 70 in newborn boys conceived by ART, at a false discovery rate (FDR) of less than 0.01 (Supplemental Tables 1 and Supplemental Table 2). Ten CpGs were differentially methylated according to conception by ART in both sexes: one on chromosome 2, eight on chromosome 6, and one on chromosome 12. The CpG on chromosome 12 is linked to the *PIWIL1* gene, while those on chromosomes 2 and 6 are not associated with any known genes.

**Fig. 3:**
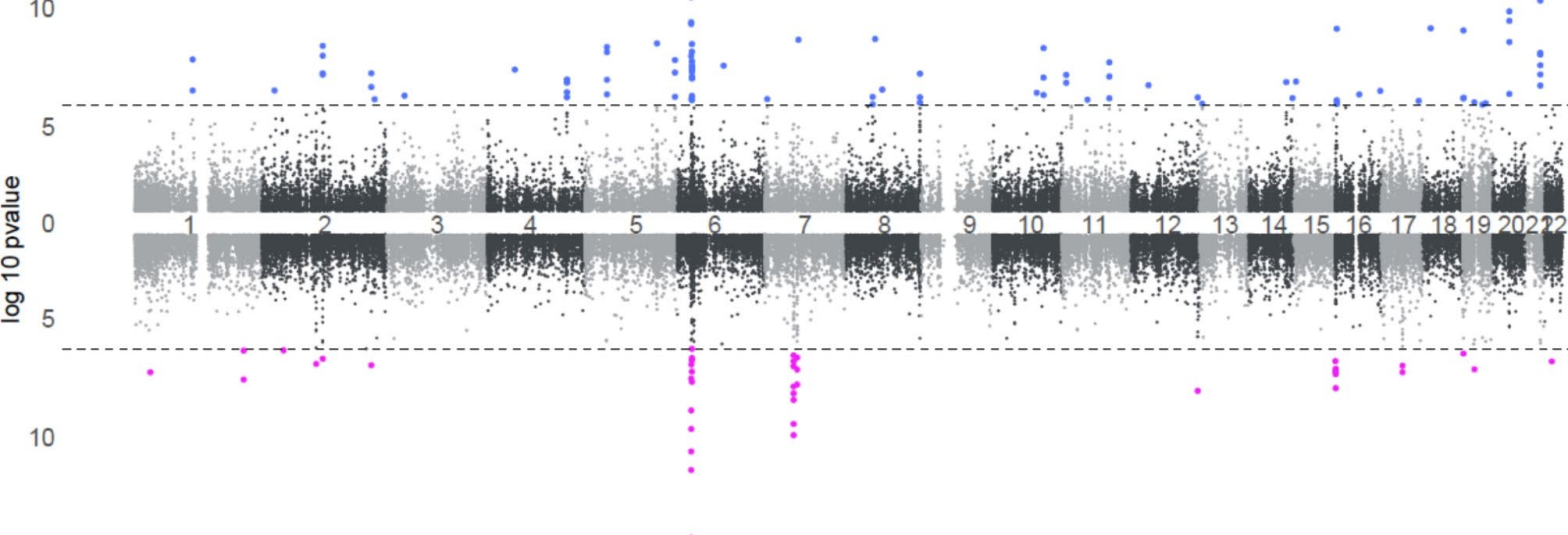
Associations between ART and epigenome-wide DNA methylation for newborn boys (blue, shown above) and girls (pink, shown below). Dashed lines indicate a genome-wide significance cut-off of FDR < 0.01, highlighting statistically significant differences.

Table 2 summarizes the known genes with differentially methylated CpGs according to conception by ART in girls and boys. Among the ART-associated CpGs sites found in girls, two CpGs mapped to the *BRCA1:NBR2* gene cluster and two CpGs with *PIWIL1.* The CpGs mapped to *BRCA1:NBR2* were significantly differentially methylated within the bidirectional promoter of *BRCA1* and *NBR2*, showing strong differences in ART-conceived newborn girls, with weaker differences observed in boys as well (Fig. 3).

**Table 2:** Number of differentially methylated CpGs in genes with significant differences (FDR < 0.01) between ART-conceived and naturally-conceived newborns.

Girls			Boys		
No. of differentially methylated CpGs per gene	No. of genes	Gene(s)	No. of differentially methylated CpGs per gene	No. of genes	Gene(s)
1	5	*LUC7L*,* STX12*,* ZNF708*,* ZNF723P*,* CDCA7P1*	1	30	*AC079610.1*,* ARPP21*,* ATP8B3*,* CCDC166*,* FAM228A*,* GS1-124K5.9*,* LOC100130274*,* LOC644669*,* NBPF24*,* OR4C13*,* PCDHGA9*,* PCDHGA8*,* PCDHGB5*,* PCDHGA1*,* PCDHGA2*,* PCDHGA3*,* PCDHGB1*,* PCDHGA4*,* PCDHGB2*,* PCDHGA5*,* PCDHGB3*,* PCDHGA6*,* PCDHGA7*,* PCDHGB4*,* PCED1CP*,* PIWIL1*,* RASL11B*,* SNORD115-45*,* TESSP1*,* XXbac-BPGBPG24O18.1*
2	4	*BRCA1; NBR2*,* PIWIL1*,* RPS6KC1*	2	2	*MTNR1B*,* APC2*
3	1	*ZNF727*	3	2	*RBM46*,* ANKRD2*
4			4	3	*NECAB3;C20orf134*,* RP11-373N24.2*
5	1	*PRR25*	5		
6	1	*LOC650226*	6	1	*GET1*

Among boys, 10 of the 70 differentially methylated CpGs were annotated to genes with functions spanning reproductive biology (*PIWIL1*, one CpG)^24^ to neurobiology (*MTNR1B*, two CpGs and *ARPP21*, one CpG)^25–27^ and cellular signaling (*APC2*, two CpGs and *NECAB3;ACTL10*, four CpGs)^25,28^.

### Interaction effects of ART-conception and sex

**Fig. 4:**
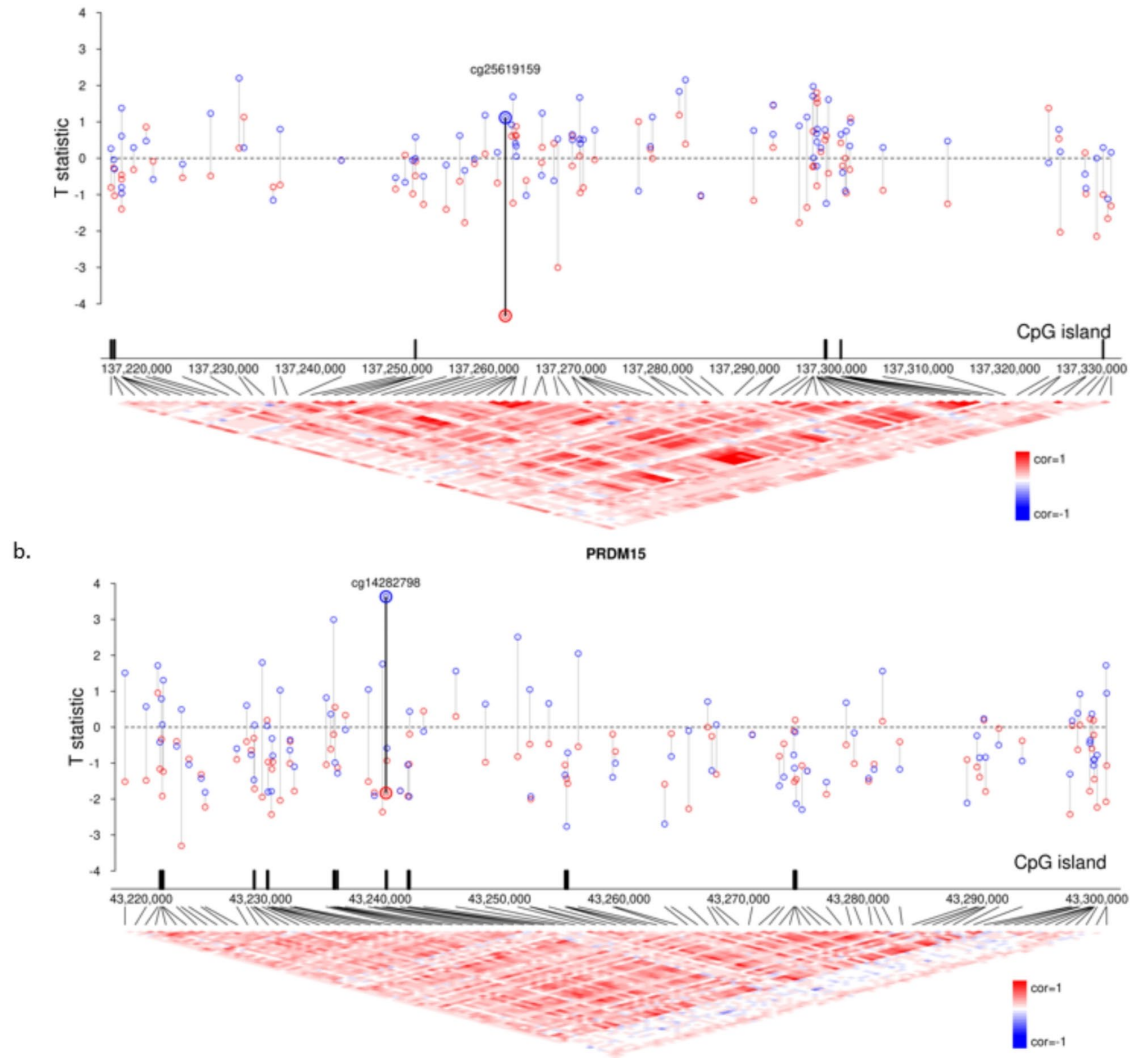
Genes with suspected interactions between ART and sex. a) RXRA (P = 9.10E-05) and b) PRDM15 (P = 1.16E-04) display the lowest ART and sex interaction P-value. The genomic coordinates are on the x-axis, and the T-statistic value for each tested CpG site on the y-axis, indicating the strength and direction of the ART-CpG association. Blue dots represent data for boys, red for girls. ​CpG islands are annotated below the x-axis. The heatmap visualizes the correlation of methylation levels between CpG sites, with red for positive correlation and blue for negative correlations.

In our analysis of the interaction effects between ART conception and sex, although several CpGs showed low *P* values, none reached an FDR < 0.01 (Supplemental Table 3). Table 3 lists the 21 genes with the lowest ‘ART-sex’ interaction *P* values, with *RXRA* and *PRDM15* showing the most notable ART-related DNAm differences. Figure 4 illustrates these differences on the T-statistic scale, highlighting the most robust evidence of a sex-interaction in the ART-related DNAm differences at cg25619159 on *RXRA* and cg14282798 on *PRDM15*.

**Table 3:** Top genes with lowest ART*sex interactive P-values.

Gene names^a^	Chr	Position^b^		Number of CpGs								
				In girls		In boys		Chisq independence test	Interaction lowest CpG	Empirical test *P* value	Interaction effect on	
											overall methylation	
				+	−	+	−	***P *** **value**	P value in gene		Effect size	P value
*SLC30A10*	1	220,091,399–220,102,528	25	9	16	13	12	0.3927	1.15E−04	0.3902	− 0.05	0.82
*RNU5F-1*	1	220,050,144–220,291,627	39	8	31	15	24	0.1363	1.15E−04	0.2663	0.10	0.43
*EVC2*	4	5,564,447–5,712,688	38	13	25	8	30	0.3049	5.42E−05	0.3492	0.02	0.63
*CPEB2*	4	15,004,091–15,068,643	24	10	14	7	17	0.5461	1.46E−04	0.4819	− 0.12	0.67
*LOC101929095*	4	15,036,233–15,430,964	48	9	39	7	41	0.7842	1.46E−04	0.7679	0.15	0.51
*LEF1*	4	108,969,808–109,091,059	53	22	31	33	20	0.0519	2.56E−05	0.1944	− 0.02	0.83
*EXOC3*	5	442,385–467,152	56	17	39	22	34	0.4276	2.32E−05	0.6081	0.08	0.31
*NRG2*	5	139,227,318–139,423,353	81	11	70	20	61	0.1101	7.96E−05	0.6500	− 0.01	0.72
*TNFRSF21*	6	47,199,817–47,278,551	40	9	31	15	25	0.2225	1.31E−04	0.2558	0.02	0.18
*CRHR2*	7	30,692,614–30,741,061	40	3	37	7	33	0.3105	7.99E−05	0.3198	− 0.01	0.71
*GGH*	8	63,929,085–63,952,370	24	7	17	4	20	0.4922	7.11E−05	0.4076	− 0.07	0.39
*FGD3*	9	95,708,321–95,798,393	45	5	40	16	29	0.0127	1.23E−05	0.0762	0.11	0.14
*RXRA*	9	137,217,063–137,332,241	86	33	53	57	29	0.0004	9.10E−05	0.0303	0.08	0.35
*SIK3*	11	116,716,214–116,970,002	89	31	58	39	50	0.2828	1.00E−04	0.8000	0.08	0.49
*LINC00365*	13	30,677,724–30,684,119	7	1	6	3	4	NA	1.24E−04	NA	NA	NA
*BRICD5*	16	2,260,190–2,262,293	7	1	6	4	3	NA	6.47E−05	NA	NA	NA
*PGP*	16	2,261,947–2,265,528	22	2	20	10	12	0.0178	6.47E−05	0.0337	− 0.12	0.91
*GALR2*	17	74,069,707–74,073,527	14	4	10	8	6	0.2519	3.54E−05	0.1785	NA	NA
*ADGRE4P*	19	6,970,783–6,990,676	3	0	3	2	1	NA	5.87E−05	NA	NA	NA
*OR7A17*	19	14,991,752–14,993,618	7	0	7	5	2	NA	6.64E−05	NA	NA	NA
*PRDM15*	21	43,218,633–43,300,824	97	14	83	36	61	0.0006	1.16E−04	0.0000	0.18	0.38

The adjusting variables were maternal age, maternal smoking, maternal BMI before pregnancy, child sex, parity, and plate ID.

^a^CpG, Illumina CpG identifier.

^b^Chromosome (chr) and position (hg19).

^c^HGNC, gene symbol.

+: positive beta estimate.

−: negative beta estimate.

NA: Chi-squared independence test did not coverge due to the small number of CpGs included.

For *RXRA*, the impact of ART on DNAm patterns varied significantly between boys and girls. While none of the 86 CpGs in *RXRA* reached statistical significance, 33 CpGs were hypermethylated and 53 wre hypomethylated in girls. In contrast, boys showed hypermethylation at 57 CpGs and hypomethylation at 29 CpGs.

For *PRDM15*, ART-related methylation differences were more consistent across sexes. Of the 97 CpGs in *PRDM15*, girls displayed hypermethylation at 14 CpGs and hypermethylation at 83 CpGs. For the same CpGs in boys, 36 were hypermethylated and 61 were hypomethylated.

We also examined the interaction between ART and sex on CpGs at the gene level by counting the occurrences of hypermethylation and hypomethylation across sexes. Only one gene, *ABCA7*, showed a marked difference DNAm associated with conception by ART between boys and girls at cg06730721 (*P* = 0.005) (Supplemental Fig. 1). However, this did not remain significant after adjusting for FDR.

### Different ART method and DNA methylation in girls and boys

When fresh embryo transfers were compared to natural conceptions, 76 statistically significant CpGs were identified in girls (*n*_natural_ = 499; *n*_fresh_ = 348) and 105 in boys (*n*_natural_ = 461; *n*_fresh_ = 391), both with an FDR < 0.01 (Supplemental Tables 4 and 5). Among these, 13 CpGs were differentially methylated in both girls and boys according to conception by fresh embryo transfer compared to natural conception. However, no sex-specific differentially methylated CpGs emerged when comparing conception by frozen embryo transfer to natural conception (Supplemental Fig. 2).

Furthermore, five statistically significant differentially methylated CpGs were identified when comparing fresh embryo transfer (*n*_fresh_ = 59) to frozen embryo transfers (*n*_frozen_ = 345) among girls, with three of these CpGs annotated to genes (one to *STX12* and two to *RPS6KC1*) (Supplemental Fig. 2b and Supplemental Table 6). By contrast, no significant CpGs were found among boys for fresh vs. frozen embryo transfers after correcting for multiple testing (FDR < 0.01).

When comparing individuals conceived by intracytoplasmic sperm injection (ICSI) to those conceived naturally, five differentially methylated CpGs were found (Supplemental Fig. 2c and Supplemental Table 7). Two of these were significant for boys (*n*_natural_ = 461; *n*_ICSI_ = 197) and three were significant for girls (*n*_natural_ = 499; *n*_ICSI_ = 158), all at an FDR less than 0.01.

Comparisons between IVF-conceived (excluding ICSI) and naturally-conceived newborns identified six statistically significant CpGs in girls (*n*_natural_ = 499; *n*_IVF_ = 348) and 11 in boys (*n*_natural_ = 461; *n*_IVF_ = 260) (Supplemental Fig. 2d and Supplemental Table 8). Among these, three CpGs on chromosome 6 were significantly methylated in both sexes, and these CpGs were also present in the broader sex-specific comparisons between overall ART-conceived and naturally-conceived newborns (Supplemental Tables 1 and 2). No significant CpGs emerged from the comparison between ICSI and IVF.

### DNA methylation and health implications for girls and boys

To investigate potential health complications associated with sex-specific variations in ART related DNAm differences, we conducted a gene-enrichment analysis focusing on genes associated with health outcomes known to vary by sex and ART^12,14,19,20^ such as birthweight, neurodevelopmental disorders, gestational age, cleft lip and palate, and childhood cancer^12,14,19,20^. Our analysis revealed an overrepresentation of ART-related DNA methylated differences in CpGs located in childhood cancer genes among girls (Supplemental Fig. 3). There was a notable overrepresentation of CpGs associated with birthweight and neurodevelopmental disorders among ART-conceived newborns, but we found no significant sex differences. Lastly, we did not find any overrepresentation of CpGs related to gestational age or cleft lip and palate in ART-conceived newborns.

## Discussion

Previous studies have shown cord-blood DNA methylation differences in newborns conceived using assisted reproductive technologies (ART) compared to those conceived naturally. However, whether these ART-related DNA methylation differences vary with children’s sex has been unknown. To our knowledge, this is the first report of sex differences in cord-blood DNA methylation variation according to conception by ART. We identified 37 differentially methylated CpGs according to ART-conception among girls, and 70 differentially methylated CpGs according to ART-conception among boys, when we used a 1% false discovery rate to account for multiple testing. Ten CpGs were differentially methylated according to conception by ART in both sexes. These sex-specific epigenetic distinctions underscore the nuanced impact of ART on the fetal epigenome, underscoring the need for further explorations into the long-term implications for sex-specific developmental trajectories in ART-conceived individuals.

Sex differences in the epigenetic regulation of *BRCA1* through DNAm have drawn significant interest due to their implications for cancer susceptibility. Although *BRCA1* mutations are recognized as pivotal contributors to breast and ovarian cancers^29^, emerging evidence suggests that DNAm patterns at the *BRCA1* transcription factor also differ between males and females^30^. In females, aberrant hypermethylation of the *BRCA1* promotor region has been linked to transcription silencing, potentially contributing to the initiation and progression of cancer^31^. There has also been some recent evidence that prenatal CpG methylation is associated with a higher risk of triple negative breast cancer among females in adulthood^32^. Given the well-established association of *BRCA1* mutations with breast and ovarian cancers in females, it is imperative to elucidate the sex-specific epigenetic mechanisms that regulate *BRCA1* in girls.

*PIWIL1*, which showed differential expression among ART-conceived boys, encodes a protein belonging to the Piwi subfamily of the Argonaute family, primarily expressed in germline cells, particularly in the testis. It plays a crucial role in maintaining genome stability by suppressing transposable elements^33^. Studies have linked *PIWIL1* with azoospermia and male infertility in mouse models^24,34^, but its role in human male infertility remains uncertain^35,36^. One study on pancreatic ductal adenocarcinoma (PDAC) cell lines demonstrated that *PIWIL1* acts as an oncoprotein by activating the APC/C E3 complex, enhancing PDAC metastasis through the targeted degradation of the cell adhesion-related protein, Pinin^37^. Another study in gastric cancer showed high *PIWIL1* expression in gastric cancer tissues, with its knockout significantly reducing cell proliferation, migration, metastasis, and tumorigenesis^38^. However, more studies are needed to establish its association with cancer in systemic models to enhance our understanding and interpretation of ART-related methylation differences in boys.

The interactive effects of ART and sex on *RXRA* and *PRDM15* underscores the complexity of their interplay and potential health impacts. *RXRA*, a nuclear receptor essential for mediating retinoid effects, is involved in immunity and repair^39^. The differential methylation observed in *RXRA* between boys and girls suggests sex-specific effects on molecular interactions between host antiviral responses and metabolic nuclear receptors, which could differently influence the immune system development and function. In mice, *Rxra* has been shown to attenuate the antiviral response by inhibiting type I interferon (IFN) production and the expression of IFN-stimulated genes, with downregulation of *RXRA* expression thought to enhance type I IFN production and host antiviral immunity^40^. While the clinical implications of this in humans are unclear, separate epidemiological studies have found that children born after ART exhibited immunological alterations^41^, as well as a higher incidence of asthma and allergies^42,43^. The connection between ART and sex-specific DNA methylation is currently poorly understood and underscores the need for further exploration into the relationship between ART, sex, and epigenetic modifications, and their impact on health outcomes in ART-conceived newborns.

Depletion of the transcription factor *PRDM15*, through genetic alterations or antisense drugs, has been shown to impede the growth of B-cell lymphoma and triggers p53-dependent apoptosis in diffuse large B-cell lymphoma^44^. *PRDM15* also plays a critical role in embryonic development and influences pluripotent stem cell reprogramming by modulating WNT and MAPK-ERK signaling pathways. This involves directly promoting the transcription of upstream regulators Rspo1 (R-spondin1) and Spry1 (Sprouty1), which are essential for maintaining the naïve pluripotency state^40^. Observations of a higher proportion of hypomethylation in girls might indicate a sex-specific sensitivity or responsiveness of this gene to certain environmental or biological cues that influence DNAm patterns, though the influence of ART-conception is not known. Further investigation into the specific regulatory elements and factors that affect *PRDM15* methylation in a sex-specific manner is warranted to understand its functional implications.

In our analysis of DNAm per gene, *ABCA7* showed DNAm differences between boys and girls. Although these differences were not statistically significant after adjusting for multiple testing, the pronounced variations observed at cg06730721 between boys and girls suggest a potential ART-sex interaction. Located on chromosome 19q13.32, *ABCA7* plays a crucial role in cellular lipid transport, particularly in the efflux of lipids, such as phospholipids and cholesterol, from cells^45^. It is highly expressed in the brain, particularly in microglia, and loss-of-function mutations in *ABCA7* are significantly associated with Alzheimer’s disease (AD), as demonstrated in genome-wide association studies (GWAS) across various populations^45,46^. Furthermore, altered methylation—both hyper- and hypomethylation—at several CpGs in *ABCA7* has been significantly associated with AD^47,48^. Notably, cg06730721 was found to be significantly hypermethylated in 9-year-old children^49^. Additionally, hypomethylation of a CpG island shore in the promotor region of *ABCA7* has been associated with late-onset Alzheimer’s compared to healthy control brains and brains of patients with dementia with Lewy bodies^50^.

It is important to note that DNA methylation, in isolation, does not definitively indicate gene expression or repression. It is worth noting that some of the reported genes showed only one differentially methylated CpG. Such findings highlight the need for caution in interpreting the functional significance solely based on the presence of a single CpG site. While DNA methylation can serve as a potential regulatory mechanism, its functional implications are context-dependent and require further investigation through RNA sequencing to determine if the observed differential methylation is accompanied by changes in gene expression levels. While our study uncovers associations between DNA methylation and ART, further investigation is necessary to elucidate the functional consequences and underlying regulatory mechanisms. In addition, reporter gene assays or chromatin conformation assays can be employed to study how the identified methylation changes affect enhancer activity or regulatory interactions with other genomic elements.

The strength of this study lies in its large sample size, which allows for a robust examination of sex-specific DNAm differences. Our analyses were further enhanced by detailed evaluations of different ART procedure and data linkage with the Medical Birth Registry of Norway (MBRN), where ART use is mandatorily reported, ensuring nearly 100% complete case ascertainment.

Specific ART procedures may introduce distinct variations in the epigenome, influenced by factors like culture conditions, hormonal stimulation, and manipulation of gametes and culture conditions. We have specifically examined cord-blood DNAm, acknowledging that it may exhibit variations distinct from other tissue types like placental tissue. We recognize that certain methylation patterns can be influenced by subtle environmental factors such as freezing/thawing cycles or changes in culture media^51,52^. Interestingly, the most sex-specific differentially methylated CpGs were observed in the fresh embryo transfers compared to natural conceptions. In contrast, comparisons between fresh vs. frozen, ICSI vs. natural conception, and IVF vs. natural conception showed fewer differences, possibly due to the smaller sample sizes in these groups, reducing statistical power. Currently in both Norway and other high-income countries, frozen embryo transfer is the most common ART procedure, favored for its ability to preserve multiple embryos and typically higher pregnancy rates^53^. This trend suggests that sex-specific differences in DNAm observed in fresh embryos may be less relevant for the majority of ART conceptions today. In this sense, the generalizability may be limited by the specific population and practices in Norway. Lastly, this study utilized the Illumina Epic Array, version 1 for DNA methylation analysis, which, while offering high coverage of CpG sites, does not provide the same depth and breadth of coverage as whole-genome bisulfite sequencing (WGBS). Consequently, methylation changes outside the targeted regions may have been missed, which could impact the comprehensiveness of our findings.

In conclusion, this study provides evidence of distinct sex differences in ART-related DNAm variations, with a limited overlap in differentially methylated CpGs between the sexes. The identification of distinct DNAm patterns in boys and girls conceived through ART compared to natural conception raises intriguing questions about how ART might affect epigenetic profiles differently based on the sex of the embryo. Further investigations into the underlying mechanisms and follow-up of this cohort for long-term consequences of these differences are essential for fully understanding the impact of ART on the subsequent health outcomes for offspring.

## Methods

This research received ethical approval from the Southeastern Regional Committee for Medical and Health Research Ethics (Approval number: #2017/1362). Written informed consent was obtained from all participants prior to their involvement in the study. All methods were performed in accordance with the relevant guidelines and regulations. The establishment of the Norwegian Mother, Father, and Child Cohort (MoBa) project and the initial data collection were conducted under license from the Norwegian Data Protection Agency and approval from the Regional Committees for Medical and Health Research Ethics^23^.

### Study population

The study population consisted of over 95,000 pregnant women enrolled in the Norwegian Mother, Father and Child Cohort Study (MoBa)^23^ from all across Norway around 18 weeks of gestation between 1999 and 2008, with partners being from 2001 onwards. As women could participate with more than one pregnancy, this resulted in over 110,000 offspring being born into the cohort. Participants are regularly followed up through self-administered questionnaires and their data are routinely linked to the Medical Birth Registry of Norway (MBRN). Blood samples were collected from the women during pregnancy and from the umbilical cord at delivery^23,54^.

The selection criteria for measuring DNA methylation levels among ART-conceived and naturally-conceived newborns has been described previously^13^. Briefly, the selection criteria were: (i) the children had to be singletons with complete records from the MBRN, (ii) their mothers needed to have completed the first MoBa questionnaire by the 17th week of gestation, and (iii) DNA samples had to be obtainable for the entire mother-father-child trios. The selection process for participants adhered to previous protocols^13^, except for six trios who opted out of the study. These trios were removed from all data sets in compliance with the General Data Protection Regulation (GDPR), leaving a total of 1,939 children in the study (Fig. 1).

### DNA methylation measurements and data processing

The processing of DNA samples for methylation values, along with data handling, quality control, and normalization, have been detailed in our previous work^13^. Briefly, DNA samples were processed at the University of Bonn’s Institute of Life & Brain Sciences, Germany, using version 1.0 of the Illumina Infinium MethylationEPIC BeadChip array^55^. The EZ-96DNA methylation-Lightning™MagPrep kit from Zymo Research (Irvine, California, USA) was used for bisulfite conversion. Raw data were acquired through Illumina’s proprietary software, Genome Studio 2011.2. Quality control was performed using the RnBeads (v2.2.0) in R (v3.5.0), processed in four separate batches^56^.

During the quality-control phase, we removed 44,210 probes^57^ that exhibited cross-hybridization and an additional 16,117 probes affected by SNPs at the last three bases. Probes with a detection p-value exceeding 0.01 were also excluded from the analysis. To address any aberrant DNAm patterns, the batches were subject to the greedycut algorithm (part of the RnBeads QC pipeline) to exclude certain samples and probes. Finally, the remaining DNA methylation signals were corrected for background noise using the ENmix.oob normalizing function^58^.

Visual inspection of the signal intensity for all samples was conducted using the output of control probes from RnBeads. If a CpG site had poor quality or a high detection p-value in one batch, it was excluded from all subsequent batches. In total, 770,586 autosomal CpGs remained in the final data set for the current analyses.

For normalization of type I and type II probes, the Beta-mixture quantile normalization (BMIQ)^59^ method from the watermelon (v1.26.0) R (v3.5.0) package^60^ was applied. Following quality control, we excluded two children due to empty plate wells, one child due to outlier values, three children due to corrupt images, and 19 children due to high background signals, resulting in a total of 1,939 children for analysis.

### ART procedures

Fertility clinics are required to report any use of ART to the Medical Birth Registry of Norway. The information includes details on specific ART procedures, such as IVF with or without ICSI, and whether embryos were transferred fresh or after being frozen-thawed. ART use was categorized as a binary variable, indicating the presence or absence of any ART (excluding inseminations). Additionally, ART was further subdivided into combinations of the subtypes mentioned above. In instances where information on the ART procedure was missing (*n* = 79), they were coded as “unknown procedure.”

### Covariates

Information on child sex, parity, and gestational age was obtained from the MBRN. Additional information was derived from MoBa questionnaires, including parental height and weight, educational attainment, and smoking status during pregnancy. Gestational age at birth was determined using ultrasound measurements when available (*n* = 1,835). For naturally-conceived children, gestational age was based on the last menstrual period (*n* = 24), and for ART-conceived children, it was calculated based on the time of embryo insertion (*n* = 82).

As part of the quality control (QC) process for the epigenetic data, it is essential to consider erroneous sex assignment where genetic sex inferred from the sex chromosomes do not match the recorded sex in MBRN. We identified discrepancies in the recorded sex of individuals and reclassified one individual initially identified as female to male and five males initially recorded as female.

### Statistical analyses

Firstly, we employed linear mixed models to estimate the association between DNA methylation at each CpG and use of ART. This involved testing the difference in DNA methylation between ART-conceived and naturally-conceived separately for boys and girls. We adjusted for maternal characteristics related to the use of ART have been shown to be associated with cord-blood mDNA, such as age^61^, smoking^62^, body mass index (BMI)^63^, and parity (strongly associated with ART)^64,65^ by including them as covariates in the linear mixed models.We also adjusted for the random effect of plate ID. For each CpG site, we excluded those with DNA methylation levels falling outside the range of median ± 5 times the median absolute deviation (MAD), where MAD is defined as the median of the absolute differences between each individual’s DNA methylation level and the overall median. As subsequent analyses, we contrasted the following groups: IVF vs. ICSI, natural conception vs. fresh embryo transfer, natural conception vs. frozen embryo transfer, natural conception vs. ICSI, and natural conception vs. IVF.

Secondly, to investigate whether the DNA methylation-ART associations differed between boys and girls, we included interactive terms of newborn sex with ART as covariates in the linear mixed models. A significant interaction term involving newborn sex and ART would indicate that the effect of ART varies between male and female newborns. We excluded outliers and conducted subsequent analyses contrasting different groups as previously described. In sensitivity analyses, additional adjustments were made for gestational age at birth, birthweight, maternal educational level, paternal age, and cell-type composition in cord-blood.

To investigate whether the distribution of DNA methylation levels differed between girls and boys, we used the KS test. All models included only newborns with available data for the variables in each respective model (see Table 1 for information on missing data). Two-sided Wald tests were used to assess the significance of the adjusted associations between ART and DNA methylation in each model. The Benjamini-Hochberg procedure^66^ was used to control for multiple testing, applying a false discovery rate (FDR) cut-off of < 0.01. We compared DNA methylation by sex within the following groups of newborns: fresh embryo transfer versus naturally-conceived newborns, frozen embryo transfer versus naturally-conceived, fresh embryo transfer versus frozen embryo transfer, IVF with ICSI versus naturally-conceived, IVF without ICSI versus naturally-conceived, and, lastly, IVF with ICSI compared directly to IVF without ICSI.

To further examine the ‘ART-sex’ interaction on overall methylation per gene, we performed a permutation analysis. We identified UCSC reference gene names listed in the Illumina’s Infinium MethylationEPIC manifest, selecting those with more than 20 CpGs for detailed analysis. For each gene, we compiled individual-level data that included ART status, sex, adjusting for the same variables as before, and methylation level for sequential CpGs, e.g., CpG1, CpG2, CpG3, etc. These data were then transformed into a ‘long-data format’. In this format, each individual’s ART status, sex, and adjusting variables are repeated across multiple rows—each corresponding to one CpG—alongside a new column that aggregates the overall methylation, stacking the methylation levels of CpG1, CpG2, CpG3, and so on, for each individual. Using this long-data format, we estimated the ART-sex interaction effect on the overall methylation. We then performed a block-wise permutation, where each block represented one individual, to generate the distribution of the test statistic, i.e., the ART-sex interaction effect, under the null hypothesis. To adjust for multiple testing, we applied the Benjamini–Hochberg procedure, setting an FDR threshold of 0.05 to capture some of the patterns that we found on the gene level analysis of the ART-sex interaction.

The analyses were performed using Stata version 16 (StataCorp) and the R statistical programming language v4.0.4 (www.r-project.org). The linear mixed models were implemented using the lme function in the nlme R package (v3.1-152).

## Electronic supplementary material

Below is the link to the electronic supplementary material.

Supplementary Material 1. Selection of study participants.

Supplementary Material 2. Differences in the distribution of genome-wide DNA methylation between ART-conceived and naturally-conceived girls (pink with dotted line), and between ART-conceived and naturally-conceived boys (blue with solid line).

Supplementary Material 3. Associations between ART and epigenome-wide DNA methylation for newborn boys (blue, shown above) and girls (pink, shown below). Dashed lines indicate a genome-wide significance cut-off of FDR < 0.01, highlighting statistically significant differences.    Fig. 4. Genes with suspected interactions between ART and sex. a) RXRA (P = 9.10E-05) and b) PRDM15 (P = 1.16E-04) display the lowest ART and sex interaction P-value. The genomic coordinates are on the x-axis, and the T-statistic value for each tested CpG site on the y-axis, indicating the strength and direction of the ART-CpG association. Blue dots represent data for boys, red for girls. ​CpG islands are annotated below the x-axis. The heatmap visualizes the correlation of methylation levels between CpG sites, with red for positive correlation and blue for negative correlations.    Supplemental Fig. 1. Permutation test for the ART association with overall DNA methylation for ABCA7 (P = 0.005), displayed on the T-statistic scale for boys and girls. The x-axis represents genomic coordinates, while the y-axis shows the T-statistic value for each CpG site, showing the strength and direction of the ART-CpG association. Blue dots represent results for boys, red dots for girls, with CpG islands annotated below the x-axis and a heatmap indicating the correlation of methylation levels between CpG sites, with red a positive and blue for negative correlations.    Supplemental Fig. 2. Quantile-Quantile (Q-Q) plot showing DNA methylation differences among ART-conceived and naturally-conceived boys and girls. It displays observed vs. expected -log P values for various comparisons:  a) ART vs. natural conception, b) Fresh embryo transfer vs. natural conception, c) Fresh vs. frozen embryo transfer, d) frozen embryo transfer vs. natural conception, e) Intracytoplasmic sperm injection (ICSI) vs. natural conception, f) ICSI vs. in-vitro fertilization (IVF), g) IVF excluding ICSI vs. natural conception. Differences in DNA methylation for boys (red) and girls (turquoise) are shown, with each subfigure corresponding to a different ART procedure comparison.    Supplemental Fig. 3. Quantile-Quantile plot of observed versus predicted –log10 P values for methylation differences associated with ART in boys and girls across CpGs related to childhood cancer, birthweight, neurodevelopment, gestational age, and cleft lip and palate. The observed –log10 P values (calculated from t statistics) are plotted against expected values, with lines indicating the background DNA methylation for all genes (light blue for boys and pink for girls) and methylation for genes enriched in specific categories for childhood cancer, birthweight, neurodevelopment, gestational age, and cleft lip and palate. They represent the background DNA methylation for all genes. The dark blue (boys) and dark red (girls) indicate DNA methylation for genes enriched for: a) childhood cancer b) birthweight on a continuous scale (in grams), c) birthweight d) Mendelian neurodevelopmental disorders vs. controls, and e) cleft lip and palate. The diagonal line indicates expected P values if there were no differences between the groups. Dots indicate the P values for a subset of CpGs that have been reported to be significant.

Supplementary Material 4

## Data Availability

Annotations for the CpGs were retrieved from the Illumina EPIC manifest file (https://support.illumina.com/array/array_kits/infinium-methylationepic-beadchip-kit/downloads.html) and the UCSC genome browser build Hg19 (https://genome.ucsc.edu/), which provide details such as CpG probe ID, probe sequence, chromosome position, and gene name. Approved gene names were sourced from the HUGO Gene Nomenclature Committee (HGNC; https://www.genenames.org).The data supporting the findings of this study can be accessed through the Norwegian Institute of Public Health (NIPH). However, they are subject to specific access restrictions due to the conditions of the original study-specific approvals. Therefore, the data are not publicly available. However, individual-level data can be accessed under restricted conditions, pending approval from Norwegian Ethical committees, provided that the applications align with the consent received. Requests for data access can be made through the NIPH website at https://www.fhi.no/en/studies/moba/. For specific inquiries regarding data access for this study, please contact Siri.Haberg@fhi.no. The data generated in this study are included in the Supplemental Information.

## Declarations

### Patient and public involvement statement

Patients and members of the public were actively involved in the design of this study. Feedback from a patient advisory group was sought to refine the research questions and methodology. Furthermore, public representatives participated in the dissemination planning to ensure the research findings are communicated effectively to the broader community.
