# Supplementary figures and images for: Sex differences in DNA methylation variations according to ART conception-evidence from the Norwegian mother, father, and child cohort study
Source: Sci Rep. 2024 Oct 2;14:22904. doi: 10.1038/s41598-024-73845-3 (PMC11447267; doi:10.1038/s41598-024-73845-3)

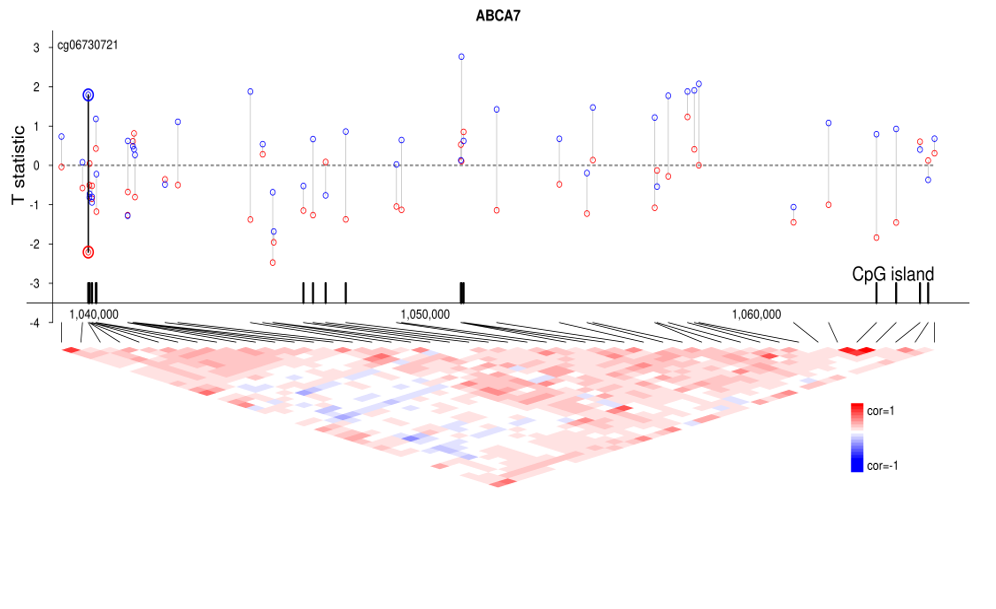

Supplement: Supplementary file 1 — Supplementary Material 1. Selection of study participants. [file 41598_2024_73845_MOESM1_ESM.png]

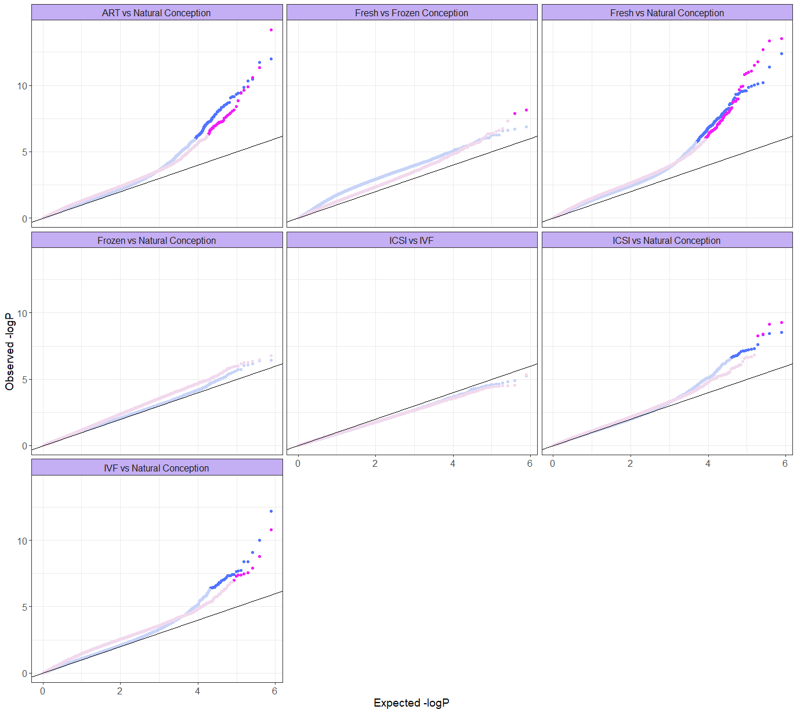

Supplement: Supplementary file 2 — Supplementary Material 2. Differences in the distribution of genome-wide DNA methylation between ART-conceived and naturally-conceived girls (pink with dotted line), and between ART-conceived and naturally-conceived boys (blue with solid line). [file 41598_2024_73845_MOESM2_ESM.png]

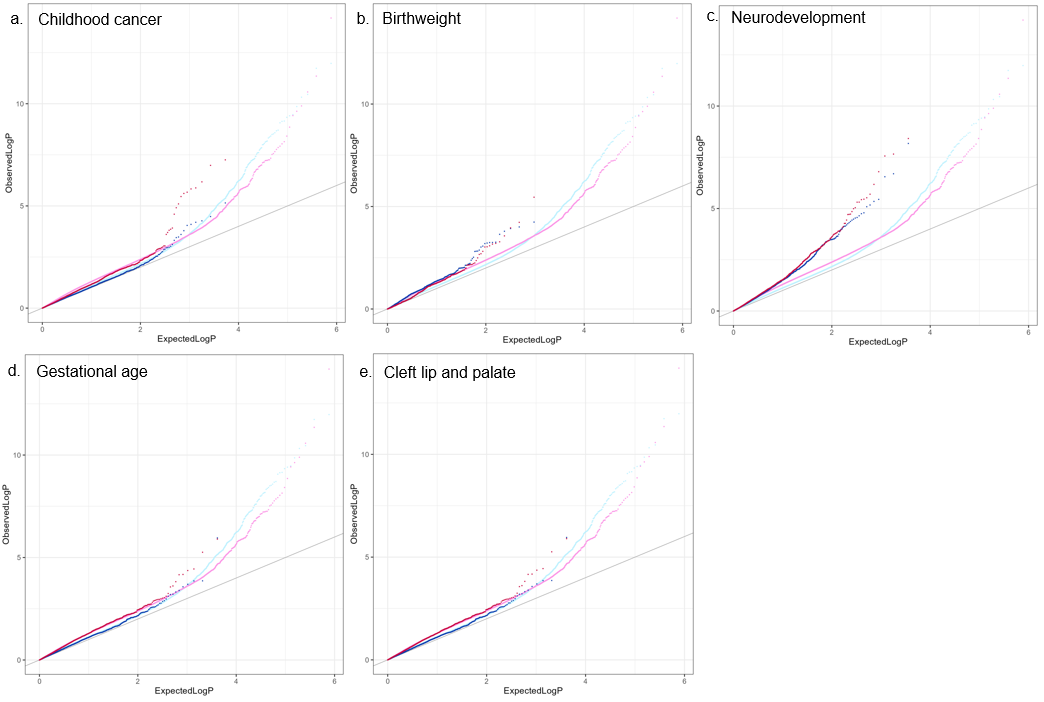

Supplement: Supplementary file 3 — Supplementary Material 3. Associations between ART and epigenome-wide DNA methylation for newborn boys (blue, shown above) and girls (pink, shown below). Dashed lines indicate a genome-wide significance cut-off of FDR < 0.01, highlighting statistically significant differences. Fig. 4. Genes with suspected interactions between ART and sex. a) RXRA (P = 9.10E-05) and b) PRDM15 (P = 1.16E-04) display the lowest ART and sex interaction P-value. The genomic coordinates are on the x-axis, and the T-statistic value for each tested CpG site on the y-axis, indicating the strength and direction of the ART-CpG association. Blue dots represent data for boys, red for girls. ​CpG islands are annotated below the x-axis. The heatmap visualizes the correlation of methylation levels between CpG sites, with red for positive correlation and blue for negative correlations. Supplemental Fig. 1. Permutation test for the ART association with overall DNA methylation for ABCA7 (P = 0.005), displayed on the T-statistic scale for boys and girls. The x-axis represents genomic coordinates, while the y-axis shows the T-statistic value for each CpG site, showing the strength and direction of the ART-CpG association. Blue dots represent results for boys, red dots for girls, with CpG islands annotated below the x-axis and a heatmap indicating the correlation of methylation levels between CpG sites, with red a positive and blue for negative correlations. Supplemental Fig. 2. Quantile-Quantile (Q-Q) plot showing DNA methylation differences among ART-conceived and naturally-conceived boys and girls. It displays observed vs. expected -log P values for various comparisons: a) ART vs. natural conception, b) Fresh embryo transfer vs. natural conception, c) Fresh vs. frozen embryo transfer, d) frozen embryo transfer vs. natural conception, e) Intracytoplasmic sperm injection (ICSI) vs. natural conception, f) ICSI vs. in-vitro fertilization (IVF), g) IVF excluding ICSI vs. natural conception. Differences in [file 41598_2024_73845_MOESM3_ESM.png]
